# Evaluation of *BRCA1* and *BRCA2* as Indicators of Response to Immune Checkpoint Inhibitors

**DOI:** 10.1001/jamanetworkopen.2021.7728

**Published:** 2021-05-07

**Authors:** Zhijun Zhou, Min Li

**Affiliations:** 1 Department of Medicine, The University of Oklahoma Health Sciences Center, Oklahoma City

## Abstract

This cohort study examines the association of *BRCA1* and *BRCA2* with tumor mutation burden and response to immune checkpoint inhibitors.

## Introduction

Immune checkpoint inhibitors (ICIs) have achieved impressive success in a subset of malignant tumors. Tumor mutation burden (TMB), programmed cell death ligand 1 expression, and deficient DNA mismatch repair are the few biomarkers known to be associated with response to ICIs. Breast cancer type 1 or 2 susceptibility gene (*BRCA1* [OMIM 113705] and *BRCA2* [OMIM 600185]) play critical roles in DNA repair. However, the roles of *BRCA1/2* alteration in tumor immunotherapy remains uncharacterized, to our knowledge. Several early phase clinical trials are examining the combination of ICIs and poly(ADP-ribose) polymerase inhibitor in *BRCA1/2* altered tumors. The role of *BRCA1/2* alteration in immunotherapy remains controversial across different tumor types. We hypothesized that *BRCA1/2* alteration is associated with TMB and may serve as a novel indicator associated with better treatment outcomes of ICIs.

## Methods

This cohort study was deemed exempt from institutional review board approval and informed consent by the University of Oklahoma Health Sciences Center ethics committee, as only deidentified human samples were used. This study is reported following the Strengthening the Reporting of Observational Studies in Epidemiology (STROBE) reporting guideline.

*BRCA* alteration status data were obtained from the cBioPortal platform.^1^ The included alteration types are missense, nonsense, nonstart, fusion, frame-shift deletion or insertion, in-frame deletion, and splice. Patients in the Memorial Sloan Kettering Cancer Center (MSKCC) cohort who received ICIs and genome sequencing were enrolled in the survival analysis.^2^ TMB was determined by normalizing the number of nonsynonymous alterations based on the sequenced genome.^2^ High TMB was defined as the top 10% of TMB in each tumor type, while the bottom 90% was regarded as low TMB.^2^

The primary outcome was overall survival (OS), started from the first day receiving ICIs. Kaplan-Meier analysis was performed to compare OS in patients with or without *BRCA1/2* alteration, and log-rank test was applied. The significance level was α = .05 for a 2-sided test. Statistical analysis was performed in SPSS version 20.0 (IBM), Prism version 5.0 (GraphPad) and R version 3.6.3 (R Project for Statistical Computing). Data were analyzed from July to November 2020.

## Results

**Figure 1.**
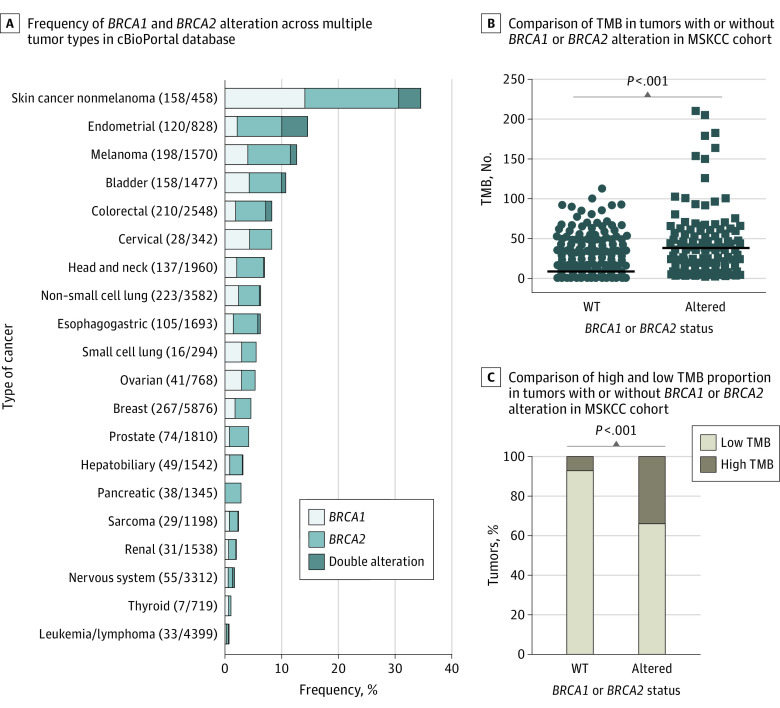
Frequency of *BRCA1/2* Alteration Across Multiple Tumors and Correlation With Tumor Mutation Burden (TMB) MSKCC indicates Memorial Sloan Kettering Cancer Center; WT, wild type. B, unpaired *t* test was applied with 2-tailed *P* value. C, Cochran-Mantel-Haenszel test was applied.

A total of 39 307 tumor samples from 37 259 patients were included in the study, including 1977 patients (5.3%) with a *BRCA1/2* alteration. Among them, 164 patients (0.4%) had double alteration, 662 patients (1.8%) had *BRCA1* single alteration, and 1151 patients (3.1%) had *BRCA2* single alteration. The prevalence of *BRCA1/2* alteration across multiple tumor types is presented in Figure 1A. *BRCA1/2* altered tumors had higher median (interquartile range [IQR]) TMB (24.59 [9.84-52.14]) than that in wild-type (WT) tumors (5.90 [2.95-10.00]; *P* < .001) (Figure 1B). A total of 49 patients (34.8%) with *BRCA1/2* alteration also had high TMB, while 1399 patients (92.0%) with WT *BRCA1/2 *had low TMB (*P* < .001) (Figure 1C).

**Figure 2.**
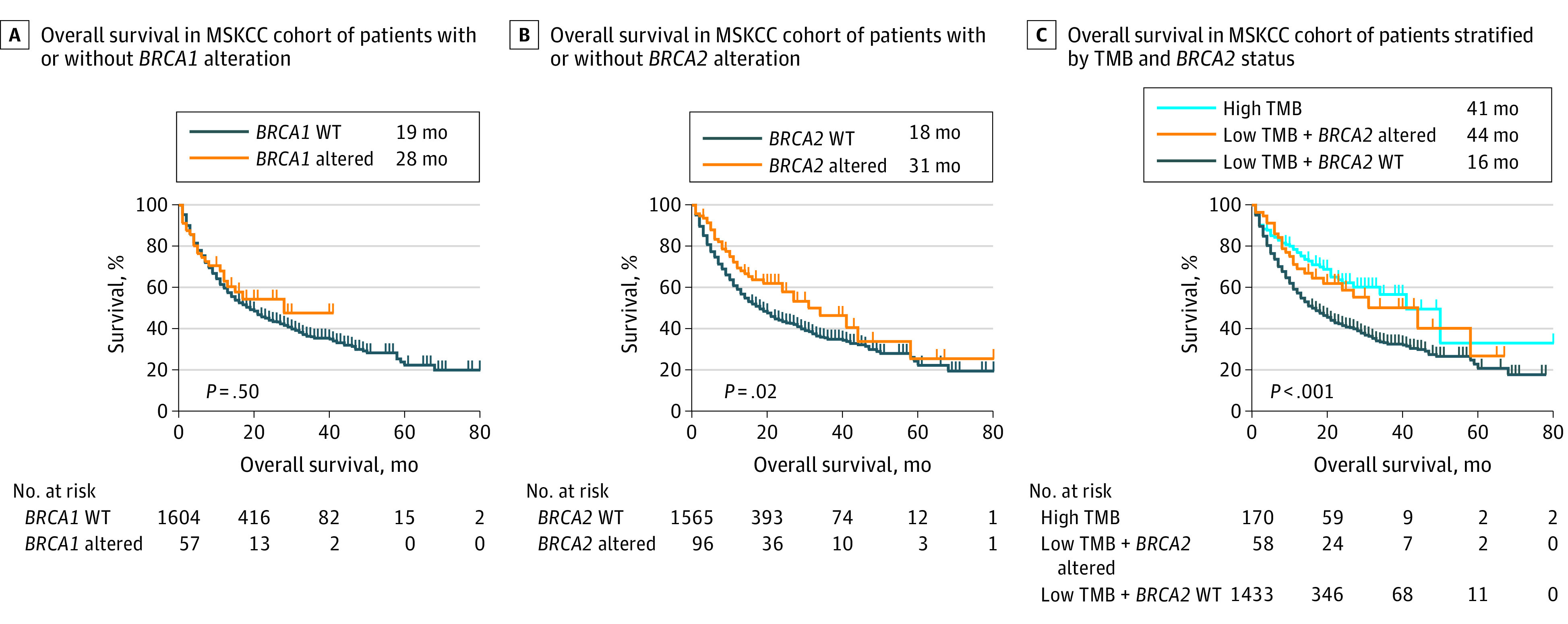
Prognostic Association of Tumor Mutation Burden (TMB) and *BRCA1/2* Alteration in Patients Receiving Immune Checkpoint Inhibitors MSKCC indicates Memorial Sloan Kettering Cancer Center; WT, wild type.

A total of 1661 patients in the Memorial Sloan Kettering Cancer Center (MSKCC) cohort who received ICIs and genome sequencing were enrolled in the survival analysis.^2^ There were 141 patients (8.5%) with *BRCA1/2* alteration in the MSKCC immunotherapy cohort. *BRCA1* alteration was not associated with OS in the MSKCC cohort (Figure 2A). Patients with *BRCA2* altered tumors had better OS than those without (median [IQR] OS, 31.0 [10.0-80.0] months vs 18.0 [6.0-58.0] months; *P* = .02) (Figure 2B). Patients with low TMB *BRCA2* altered tumors had comparable OS with patients with high TMB tumors (median [IQR] OS, 44.0 [10.0-67.0] months vs 41.0 [13.0-80.0] months), and both groups had better OS than patients with low TMB WT *BRCA2* tumors (median [IQR] OS, 16.0 [6.0-57.0] months; *P* < .001) (Figure 2C).

## Discussion

The findings of this cohort study suggest that *BRCA2* alteration in combination with TMB was a potential biomarker associated with response to ICIs. *BRCA1*/2 altered tumors have shown enhanced immunosurveillance in several preclinical studies, but their correlation with ICI treatment outcomes remains uncharacterized. Early phases of randomized clinical trials have shown promising results of combining poly(ADP-ribose) polymerase inhibitor with ICIs in *BRCA1/2* altered tumors.^3,4^

This study has some limitations, such as that the role of *BRCA2* alteration in immunotherapy in specific tumor type warrants further study. This study includes both pathogenic and undefined variants, which may result in higher alteration rates. Previous studies from our group and others have reported that CXC chemokine receptor 2 (*CXCR2*) plays critical roles in tumor immune evasion and progression.^5,6^ Further studies are warranted to pinpoint the specific tumor types that are responsive to ICIs when *BRCA2* is altered, and to verify whether the addition of *CXCR2* inhibition can further improve survival in these patients.
